# Patient and clinician perspectives on clinical trials in transplant failure: a qualitative interview study

**DOI:** 10.1186/s13063-026-09732-1

**Published:** 2026-05-11

**Authors:** Bonnie Venter, Jane Noyes, Lucy E. Selman, Catherine Exley, Siân Griffin, Alan Hancock, Leah McLaughlin, Barnaby Hole, Pippa K. Bailey

**Affiliations:** 1 Bristol Medical School: Population Health Sciences, University of Bristol, Bristol, BS8 2PS UK; 2 School of Health Sciences & Wales Kidney Research Unit, Bangor University, Bangor, LL57 2EF UK; 3 Population Health Sciences Institute, Newcastle University, Newcastle, NE2 4AX UK; 4 University Hospital of Wales & Wales Kidney Research Unit, Cardiff & Vale University Health Board, Cardiff, CF14 4XW UK; 5 Patient co-investigator working with the University of Bristol, Bristol, UK; 6 North Bristol NHS Trust, Bristol, BS10 5NB UK

**Keywords:** Kidney transplant failure, Graft failure, Qualitative research, Clinical trials, Equipoise, Barriers and facilitators to research

## Abstract

**Background:**

Transplant failure is associated with increased morbidity, mortality and poor quality of life. There is limited evidence to inform treatment decision-making when and after kidney transplants fail: randomised controlled trials are needed. We investigated the feasibility and acceptability of clinical trials into the management of kidney transplant failure, and explored research facilitators and barriers.

**Methods:**

We undertook in-depth interviews with adults (aged ≥ 18 years) who had failing kidney transplants or had experienced kidney transplant failure, their relatives/friends, and healthcare professionals (HCPs). Participants were recruited from three UK NHS hospitals. Iterative purposive sampling of patient participants was undertaken, aiming for diversity with respect to personal characteristics, the type of transplant received (living/deceased-donor), whether the transplant was failing or had failed, and current kidney disease treatment. Purposive sampling of HCPs ensured diversity in sex, age, ethnicity, and role. Family and friends were recruited via posters and snowball sampling. Interviews investigated perspectives on clinical trials at the time of transplant failure. Sample size was determined by reaching theoretical saturation. We undertook inductive analysis according to constructivist grounded theory.

**Results:**

Forty-one participants (17 HCPs/15 patients/9 relatives or friends) were interviewed. We identified the following categories:

Facilitators to clinical trials: analytical thinking

i. Recognition of the lack of evidence—individual and community equipoise
ii. Motivation to advance stratified and personalised medicine

Challenges to clinical trials: cognitive biases and heuristics

iii. Status quo bias—preference for existing treatment regimens
iv. Loss and risk aversion—fear of harm from research
v. Experiential learning and the availability heuristic—decisions based on recent or memorable cases

These 5 categories were considered within the core category of ‘Personal reasoning and clinical gatekeeping’.

There was strong support for immunosuppression reduction trials, with stratification of people according to clinical and immunological subtypes. HCPs and patients supported trials *after* transplant failure but were reticent to engage in trials as a transplant was failing.

**Conclusions:**

Acknowledged individual and community equipoise appear to support trial delivery and recruitment. Umbrella trials may be needed to evaluate therapies in multiple patient subgroups.

**Supplementary Information:**

The online version contains supplementary material available at 10.1186/s13063-026-09732-1.

## Background

Kidney transplantation is associated with better survival and quality of life compared to dialysis therapies [1, 2], but kidney transplants rarely function for a recipient’s lifetime. The USA and UK data suggest that approximately 20% of living-donor kidney transplants (LDKTs) and 25–30% of deceased-donor kidney transplants (DDKT) fail within 10 years of transplantation [3, 4]. There is no international consensus on the definition of ‘a failing transplant’ or ‘a failed transplant/transplant failure’ [5]. There is broad agreement amongst the British Transplantation Society [6], the American Society of Transplantation [7] and a Kidney Disease: Improving Global Outcomes (KDIGO) conference [5] that a kidney transplant is ‘failing’ if its function is declining and it is anticipated that the person with the transplant will require alternative treatment within 12 months [6]. In this study we adopt this definition of a failing transplant. Whilst there is agreement that dialysis initiation or retransplantation indicates transplant failure, this fails to capture individuals who die with a failed transplant before one of these treatments is started, or those who opt for conservative kidney management (CKM). In this study, we defined transplant failure as indicated by initiation of another kidney replacement therapy (either dialysis or retransplantation) or clinical documentation of transplant failure with CKM as the current treatment.

Kidney transplant failure is associated with a high risk of morbidity [8] and mortality [9, 10]. When a transplant is identified as failing, multiple decisions are required regarding the recipient’s current and future treatment options. A 2022 KDIGO Controversies Conference highlighted the lack of evidence on which to base these decisions [5, 11]. The conference identified the management of people with transplant failure as an international research priority [12, 13], and proposed key research targets including (i) determining prognosis and kidney failure trajectory, (ii) management of immunosuppression, (iii) management of psychological effects and medical complications in kidney transplant recipients, and (iv) listing for retransplantation and/or return to dialysis, including the indication for and timing of surgical procedures such as transplant nephrectomy.

Randomised controlled trials (RCTs) are urgently needed to address gaps in our understanding of the optimal management of failing transplants and kidney transplant failure. Our study aimed to investigate the feasibility and acceptability of future clinical research in this area, by investigating facilitators and challenges to research. Patient and healthcare professional (HCP) views on clinical trials to improve care have not been investigated [14, 15]. A lack of clinician equipoise has been cited as a barrier to recruitment to RCTs in all fields [16–18]. Our study aimed to explore patients’ and HCPs’ perspectives on clinical trials at the time of transplant failure, including investigating clinical equipoise, support for recruitment and randomisation, and patient perspectives on participation. The understanding gained through this qualitative research should aid the design and delivery of much-needed RCTs.

## Methods

This study focussed on perspectives towards clinical trials as transplants are failing and at/after the time of transplant failure: it was part of a larger project investigating the experiences of living with and managing transplant failure (protocol published [19]).

### Setting

Individuals were invited from three purposefully selected geographically disparate UK hospitals (two transplant units and one non-transplanting referral unit). One hospital had a specific clinic for recipients with failing transplants; in the second, recipients with failing transplants were seen in both general transplant and ‘low GFR (glomerular filtration rate)’ clinics; and in the third, patients were seen in general renal/transplant clinics.

### Eligibility

Adults from the following groups *were eligible*:

1. Individuals with
  - A failing kidney transplant. This was defined as declining transplant function, indicated by rising creatinine or falling eGFR/creatinine clearance, with a clinician’s prediction that they will require alternative treatment (e.g. dialysis, retransplantation, or CKM) within 12 months.

OR

b. A transplant that had failed in the last year. Transplant failure was defined by initiation of another kidney replacement therapy (either dialysis or retransplantation) or clinical documentation of transplant failure with CKM as the active treatment.

2. Family or close friends of individuals who met Group 1 criteria.
3. HCPs (nephrologists/transplant surgeons/pharmacists/nurse specialists/psychologists). UK specialist pharmacists and transplant specialist nurses deliver clinics, undertake pre- and post-transplant clinical education, and support transition after transplant failure. Renal nurses who work in the field of nephrology (e.g. dialysis delivery, conservative kidney management, paediatric-adult transition) but not directly with post-transplant patients were also invited to participate.

### Sampling

Iterative purposive sampling of patient participants was undertaken, aiming for diversity with respect to the Cochrane Equity Framework’s PROGRESS-Plus characteristics [20], the type of transplant received (living/deceased-donor), whether the transplant was failing or had failed, and current kidney disease treatment. Purposive sampling of HCPs ensured diversity in sex, age, ethnicity, and role. Sample size was determined by reaching theoretical saturation [21] when new data were no longer adding further insights to the categories [22]. A previous review of 83 studies found that a sample of 20–30 usually allows theoretical saturation in grounded theory studies [23, 24].

### Recruitment

Individuals were invited by local collaborators. Posters were displayed in clinical areas at participating sites. Family and friends were recruited via posters in hospital outpatient areas, and ‘snowball sampling’ via patients. HCPs were invited to participate by local collaborators and study investigators but contacted BV directly to participate. Patient and family/friend participants were given a £20 voucher as a token of thanks for participation, but HCPs were not.

### Data collection

Participants took part in an in-depth, semi-structured interview conducted over a virtual platform or telephone (except for one in-person interview) by BV. BV is an experienced qualitative researcher, female, with a background in living kidney donation policy. BV had no previous relationship with participants. Participants were informed that BV was not an HCP. All participants provided written consent. Demographic data were requested. Flexible topic guides [19] were used which evolved as interviewing progressed. Discussions focussed on perspectives on clinical trials as transplants fail, and at/after the time of transplant failure, and explored views towards clinical trials of immunosuppression minimisation, transplant nephrectomy, and participant research priorities.

### Analysis

Interviews were audio-recorded, transcribed verbatim, anonymised, and analysed using the iterative, inductive method described in constructivist grounded theory [22, 25–27], facilitated by NVivo [28]. Line-by-line coding was used to identify ideas and concepts of interest, followed by focussed coding in which initial codes were reduced to a set of selected central codes. Constant comparison was used to compare codes and group codes together to form categories. Relationships between categories were identified and the core category or central concept to which concepts related was identified [29]. Theoretical coding followed in which relationships between categories were investigated to relate the main categories to the core categories and explanatory theory. Memoing was used to capture analytic thinking, document decisions, and trace the development of categories. Summary memos on content were written by BV after each interview and shared with PKB. Analytic memos with questions of the data and thoughts on theoretical sampling were written by BV and PKB independently during coding. BV and PKB met fortnightly to discuss interviews, coding, developing theory, and sampling. Initial findings were discussed with co-investigators and five patient advisors. A reflexivity statement is provided (Supplementary material – Reflexivity). All transcripts were coded by BV and PKB. Findings were discussed with co-investigators and five patient advisors, but not participants.

The study received NHS Research Ethics Committee approval [23/WM/0020]. Reporting followed COREQ guidance [30].

## Results

Forty-one participants were interviewed (17 HCPs/15 patients/9 relatives or friends). Participant characteristics are presented in Table 1. Interviews ranged from 29 to 82 min. Table 2 presents the theoretical categories. Table 3 presents illustrative quotes.

**Table 1:** Participant characteristics

**Characteristics**	**Patients** ***n* = 15 (%)**	**Family/friends** ***n* = 9 (%)**	**HCPs** ***n* = 17 (%)**
**Sex**			
Female	8 (53)	9 (100)	10 (59)
Male	7 (47)	0	7 (41)
**Age group (years)**			
18–29	0	0	0
30–39	1 (7)	1 (11)	1 (6)
40–49	2 (13)	1 (11)	7 (41)
50–59	5 (33)	3 (33)	9 (53)
60–69	3 (20)	4 (44)	0
70–79	4 (27)	0	0
**Ethnicity**			
White	12 (80)	8 (89)	14 (82)
UK minority ethnicity: all other ethnic groups combined^a^	3 (20)	1 (11)	3 (18)
**Marital status**			
Single	3 (20)	1 (11)	
Married/long-term partner	7 (47)	6 (67)	
Other (divorced; widowed/bereaved)	4 (27)	1 (11)	
Not disclosed	1 (7)	1 (11)	
**Participant subgroups**			
Individuals living with a failing/failed transplant			
• *Transplant type (current or most recent transplant)*			
o *Living-donor kidney transplant*	4 (27)		
o *Deceased-donor kidney transplant*	11 (73)		
• *Current treatment*			
o *Receiving dialysis*	6 (40)		
o *Failing transplant, not currently receiving dialysis*	7 (47)		
o *Conservative kidney management*	2 (13)		
Family members or close friends^b^ • *Spouse/Partner* • *Parent* • *Adult child* • *Friend*		6 (67) 1 (11) 1 (11) 1 (11)	
Healthcare professionals			
• *Consultant nephrologist*			8 (47)
• *Consultant transplant surgeon*			2 (12)
• *Renal pharmacist*			2 (12)
• *Clinical psychologist*			1 (6)
• *Transplant specialist nurse*			2 (12)
• *Renal nurses*			2 (12)
**Patient and family—highest level of education**			
Secondary school	3 (20)	3 (33)	
Vocational/technical training	5 (33)	2 (22)	
University undergraduate degree	1 (7)	3 (33)	
University postgraduate degree	2 (13)	0	
Not disclosed	4 (27)	1 (11)	
**Patient and family—employment status**			
Unemployed	1 (7)	1 (11)	
Full or part-time employment	5 (33)	4 (44)	
Retired and other (e.g. student, homemaker)	7 (47)	4 (44)	
Not disclosed	2 (13)	0	

^a^All other ethnic groups combined includes: Asian/Asian British; Black/African/Caribbean/Black British; Other; Mixed/multiple ethnic groups. Information on subgroups omitted due to small numbers risking identification

^b^Three family members were living kidney donors to a person with transplant failure

**Table 2:** Categories

Core category: personal reasoning and clinical gatekeeping	
Facilitators to clinical trials: analytical thinking	Recognition of the lack of evidence, with individual and community equipoise
	Motivation to advance stratified and personalised medicine
Challenges to clinical trials: cognitive biases and heuristics	Status quo bias—preference for existing treatment regimens
	Loss and risk aversion—fear of harm from research
	Experiential learning and the availability heuristic—decisions based on recent or memorable cases

**Table 3:** Theoretical categories and illustrative quotes

Categories	Illustrative quotes
**Recognition of the lack of evidence, with individual and community equipoise** **(Exclusively identified in HCP interviews)**	*The way that we do things at the moment is that we continue a low dose CNI [calcineurin inhibitor] but to my knowledge, that’s not evidence based so … you need randomised controlled trials to show you the best way to proceed don’t you? (HCP-016: male/30–39 years/consultant transplant surgeon)*
	*I’m a bit conservative about people going into randomised trials but yes, I would consider that. I’m just thinking about the circumstance where I really believe that stopping one [immunosuppressive] was better than another but actually I don’t think I know which is the whole point about doing the trial, so yes, I’d probably say yes to that. (HCP-015: female/50–59 years/consultant nephrologist)*
	*I’m aware that such and such unit does this and such and such unit does that … and I think there is inequity, because there is this lack of evidence and I think a better evidence base would mean that patients get the same care. (HCP-005: female/40–49 years/Renal pharmacist)*
	*I think patients might find it more difficult to be randomised to surgery or no surgery to be honest, but that’s not a reason not to try if we think that we genuinely don’t know. I don’t know for sure but I think the practice varies worldwide, I think in some places transplants are more likely to be removed, in our centre or in the UK it certainly seems less common for that to happen … we still don’t know the answer so I think doing a trial is a good idea. (HCP-012: male/40–49 years/consultant nephrologist)*
	*I find a lot of difficulty for example about whether or not stopping steroids with transplant patients is the right thing to do or not. It’s generally deemed to be not but there’s so many different things, when people have cancer do you… I was talking to my mate the other day, do you stop some of their immunosuppressant drugs, do you reduce them, and he says, well, individualise it. And I’m like, well there’s some guidance… so there’s professional variability. (HCP-004: male/40–49 years/consultant nephrologist)*
	*I: Then when the transplant is failing, do patients require any changes, for instance to their medication?* *R: Sometimes we do change their immunosuppressant medication, but if you lined up the 14 consultants, or 15 consultants I work with, they’d all have very different views. We as transplant specialist nurses wouldn’t necessarily ourselves change that medication; it would be the consultant in charge of that particular patient. (HCP-008: female/50–59 years/transplant specialist nurse)*
**Motivation to advance stratified and personalised medicine** **(Exclusively identified in HCP interviews)**	*I mean, if we had patients who were not going to be relisted for transplantation then I would be more comfortable with that [comparing stopping a CNI or an anti-proliferative] as a prospective assessment of the development of sensitisation, but I think if it was all patients including those who might be going back on the list I wouldn’t be comfortable with something as black and white as stopping either one [immunosuppressive] or the other. (HCP-010: female/50–59 years/consultant nephrologist)*
	*We’ve got a more heterogeneous recipient pool now. Transplantation used to be young and middle-aged people but now it’s young people all the way up to, well there’s 80 year-olds in the clinic … so I think we need to have data for all the people … cause if you have a kidney transplant in your early 70 s and it lasts eight years and then you’re 80 years old and your kidney transplant’s failing then you might go onto conservative care, that’s a very different patient to your 18 year-old or your 25 year-old or a 30 year-old with a failing transplant, you know? … And I think the population’s growing much more diverse and people are having two or three or four transplants and then they’re much older…we have to reflect that in our research… (HCP-014: female/50–59 years/consultant nephrologist)*
	*If you had a failed transplant in situ, you are still at risk of rejection. I don’t know how you could randomise somebody to that [transplant nephrectomy] … I just don’t think that would be practical. I think there’s too many individual variables that would need to be accounted for within a patient demographic to be able to put into a trial. (HCP-005: female/40–49 years/renal pharmacist)*
	*We have evidence and we should be practising evidence based medicine, but there are situations where the evidence or whatever the treatment that’s suggested by the evidence as being the best isn’t the best because of a patient factor, for example. (HCP-016: male/30–39 years/consultant transplant surgeon)*
	*I think that maybe a trial looking at whether it’s right to biopsy more people with a failing transplant, cause I think a biopsy really guides you on your immunosuppression, whether to keep one or lose one or add another one, I think it really guides you, so whether there would be like a protocol biopsy at like a GFR of 20 or something like that, that maybe people should have that. (HCP-014: female/50–59 years/consultant nephrologist)*
**Status quo bias—preference for existing treatment regimens**	*Yes, I – um, no I probably wouldn’t be too keen on that because, you know, there’s a good phrase, which is if it ain't broke, don’t fix it you know, so if that kidney’s failed, but it’s not causing any problems you know, leave it alone. [laugh] (HCP-016: male/30–39 years/consultant transplant surgeon)*
	*I think generally, cause I feel like I’m so sort of, uh my body seems to be in a very fragile kind of state really, you know, it got affected by that diet for example … it took me months to get over it, I’m a bit worried doing anything like that [a clinical trial] would cause me to lose balance again kind of thing.” (PT-008: male/50–59 years/White/failing DDKT)*
	*I think it’s a valid question to be answered [how to manage immunosuppression at the time of transplant failure] and I don’t know the answer and I have clinical inertia… (HCP-004: male/40–49 years/consultant nephrologist)*
	*[Evidence that changes are happening to medications in clinical practice i.e. status quo is change]* *They keep changing it because firstly I think they took out all the kidney rejection tablets …and they gave me more steroids but when they saw the kidney starting to pick up, they started putting me back to the kidney tablets slowly. (PT-007: female/60–69 years/UK minority ethnicity/failed DDKT now receiving conservative kidney management)*
	*[Evidence that changes are happening to medications in clinical practice i.e. status quo is change]* *I’d been on certain medications for so long, but they were possibly now, rather than doing good, they were possibly doing harm so they’ve changed my meds and things like that. (PT-002: female/50–59 years/White/failing LDKT)*
	*Participant: Yes, me medication’s been changed since I have gone onto dialysis, they have taken me off the mycophenolate* *Interviewer: Did they explain to you why?* *Participant: Erm well when I read about it they should have kept me on it, but I can’t remember what they said, they did give me something at the time, but I ended up researching it meself and when I researched it they said it would have been better for me to stay on it… but who am I to say, you rely on your consultations, the registrars. (PT-006: male/70–79 years/White/failed LDKT now receiving dialysis)*
**Loss and risk aversion—fear of harm from research**	*I’ve always volunteered for various things, but I generally draw the line at anything which goes into my body. Now, there has been the case that if you're dying and you were offered a groundbreaking treatment as a last resort, because you have no other options – do you see what I’m saying? (PT-005: male/50–59 years/White/failed DDKT*^*1*^* now receiving dialysis)*
	*I wouldn’t mind taking part in clinical trials, unless it meant mucking about with all my medicine, that’s the only problem because they could shorten my life. (PT-001: female/70–79 years/White/failed DDKT now receiving conservative care)*
	*I think [antimicrobials at the time of transplant failure] that’s unlikely to do them any harm, I think that’s the least controversial of all of them, so yeah, that would be fine by me. (HCP-015: female/50–59 years/consultant nephrologist)*
	*I don’t think I would want to do [minimise or stop immunosuppression when a transplant is failing] ‘cause I think that that could be quite significantly impact things and make it worse, to be honest. So yes, I think I would be a bit reluctant to. [laugh] I guess if it’s stopping altogether I would probably be a bit more nervous about doing that, to be honest. (HCP-018: female/40–49 years/consultant nephrologist)*
	*We need to speak people’s language and try and explain in a way that they would understand why it’s a benefit… (HCP-011: female/40–49 years/renal nurse)*
**Experiential learning and the availability heuristic—decisions based on recent or memorable cases** **(Exclusively identified in HCP interviews)**	*No [to an RCT of transplant nephrectomy vs transplant remains in situ after failure]. Done it before, dangerous, horrible operation and can make people more difficult to transplant again. I was involved in a study where we were trying to gather some information to get an NIHR grant to specifically look at what happens with transplant failure. We had some data ourselves at [Hospital name redacted] … and we started to take kidneys out of people as part of ‘let’s see what happens’. Quite a lot of badness happened, so, I think for the right person, sure we can take the kidney out, but doing it as an elective procedure, I wouldn’t be happy to recommend that … It's never been proven, so a study would be needed. But I think there is sufficient experience and anecdote that I don’t think I have equipoise in my decision-making. (HCP-002: male/50–59 years/consultant nephrologist)*
	*Interviewer: Would you be willing to recruit patients to a study comparing the CNI stopping with the anti-proliferative stopped at the time of transplant failure?* *Participant: No. I’m comfortable with stopping the anti-proliferative, and in fact that’s what we do because they tend to get bone marrow suppression when their kidney function is low, so you can have anaemia that’s difficult to treat, they can get infections, and it doesn’t seem to be particularly good at prevent the development of donor specific antibodies, but there are a few publications with CNI withdrawal and that seems to be much more strongly associated with the development of DSAs. I mean, if we had patients who were not going to be relisted for transplantation then I would be more comfortable with that as a prospective assessment of the development of sensitisation, but I think if it was all patients including those who might be going back on the list I wouldn’t be comfortable with something as black and white as stopping either one or the other.” (HCP-010: female/50–59 years/consultant nephrologist)*
	*Some people need their kidneys out and some people don’t. So some people need their kidneys out, they’re infected, they’re causing inflammation, it means they get anaemic and they have immunosuppression medicine they don’t need to have, cause the kidney’s not working but they’ve still got the side effects of immunosuppression … So there’s people who are fine having a kidney left in them and there’s people who aren’t fine to have a kidney left in them, so there’s not much of a grey area I wouldn’t say. (HCP-014: female/50–59 years/consultant nephrologist)*
	*I think there are lots of complications involved in removing a kidney transplant. It’s not a – it’s not just “take it out”. It’s not straightforward surgery. So I think, I think that one I would … I wouldn’t be for it necessarily. (HCP-019: male/40–49 years/consultant transplant surgeon)*

Although family and friend participants were asked about their views on research priorities and their views on research participation, none felt able to comment, suggesting that they did not perceive research as potentially focussing on their needs. Our parallel analysis (under review) found that family members felt like bystanders and excluded from clinical consultations, so this may explain their feelings of being unable to comment on research. Presented findings reflect the perspectives of HCPs and patients.

### Personal reasoning and clinical gatekeeping

Personal reasoning and clinical gatekeeping emerged as the core category. HCPs emerged as gatekeepers to research, their attitude to a trial determining their willingness to invite patients and support recruitment. Facilitatory attitudes towards RCTs appeared to result from deliberate, analytical thinking with conscious attention to variation in practice, and an awareness and assessment of the existing research, or lack thereof. In contrast, reported barriers to clinical trials at the time of transplant failure appeared to result from cognitive biases, heuristics and experiences rather than analytical thinking. Several HCPs recognised this dissonance, in which they expressed a lack of support for a potential trial based on their clinical experience and accepted centre practice, whilst acknowledging the community equipoise and lack of robust/empirical? evidence for their perspective.

### Facilitators to clinical trials: analytical thinking

#### Recognition of the lack of evidence, with individual and community equipoise

This category was exclusively identified in data from HCPs. HCPs described both individual and community equipoise regarding the treatment of transplant failure as facilitators for conducting RCTs. One HCP described personal conservatism with respect to patients going into RCTs in general but explained that strong personal uncertainty regarding the best management of transplant failure meant she would support RCTs in this area. Equipoise was attributed to a lack of current evidence to guide the management of failing transplants, rather than uncertainty regarding the quality of existing evidence. Two HCPs described national and international variation in practice, which they attributed to a lack of evidence resulting in community equipoise.

HCPs across the multidisciplinary team described being uncertain about the management of immunosuppression at the time of transplant failure and every HCP described support for research into immunosuppression management after transplant failure. Most HCPs described individual equipoise about how to manage immunosuppression when a transplant is failing and when it has failed. However, there were differing degrees of uncertainty and differing degrees of individual equipoise. Some HCPs, whilst still expressing some uncertainty, expressed beliefs about which approach to medication they perceived as better. Evidencing community equipoise, individual HCPs had opposing beliefs regarding immunosuppression management at the time of transplant failure. Some HCPs believed that the calcineurin inhibitor (CNI) immunosuppressive medication should be stopped whilst others believed the anti-proliferative immunosuppressive should be stopped:

‘it needs to be perhaps removal of the anti-proliferative and minimization of the CNI…that’s what we do quite a lot…’ (HCP-010: female/50–59 years/consultant nephrologist)

‘…CNIs potentially are a lot more potent. So if you’re going to get someone off something and improve their longer-term outcomes, it should probably be the more potent of the two.’ (HCP-005: female/40–49 years/renal pharmacist)

Whilst most HCPs described a degree of equipoise regarding the optimal management of immunosuppression, they appeared to lack individual equipoise regarding transplant nephrectomy following transplant failure. HCPs appeared to hold clear beliefs regarding absolute indications for transplant nephrectomy (infection/refractory anaemia). HCPs suggested outside these indications that the risks of transplant nephrectomy outweighed potential benefits. HCPs described how this certainty would mean potential randomisation to non-surgery would not be supported. However, despite a lack of individual equipoise, HCPs did recognise community equipoise: one HCP described variation in international practice and a lack of ‘proof’ that transplant nephrectomy causes more harm than leaving in situ.

#### Motivation to advance stratified and personalised medicine

This category was exclusively identified in data from HCPs. HCPs described the transplant population as heterogeneous with respect to age, underlying primary disease, number of transplants, and treatment-goals depending on life-stage and life-expectancy at the time of transplant failure. As indicated, the majority of HCPs supported clinical trials as the gold standard, but several HCPs indicated that a stratified approach was required, with interventions tailored to and evaluated in specific patient subgroups. A key stratifying factor mentioned by several HCPs was whether an individual was suitable for retransplantation. HCPs expressed the greatest comfort in undertaking immunosuppression minimisation trials in people who were not candidates for retransplantation. One HCP suggested prospective observational research to determine which individuals might benefit from continued immunosuppression and which might benefit from minimisation/withdrawal. Several HCPs stated the need for trials evaluating the role of diagnostic investigations (e.g. biopsies) to guide and personalise immunosuppression management.

### Challenges to clinical trials: cognitive biases and heuristics

#### Status quo bias

This category was identified in data from both HCPs and patients. One HCP used the term ‘clinical inertia’ to describe their current practice, suggesting that in the absence of evidence to guide management, there was a tendency to leave things unchanged, implying that a status quo bias informs current clinical practice. The status quo bias was evidenced by another HCP’s comment ‘if it ain’t broke, don’t fix it’ (HCP-016: male/30–39 years/consultant transplant surgeon) with respect to surgical nephrectomy after transplant failure.

Overall, patient participants described wanting to take part in research in general terms to help other patients and ‘the whole area to progress’ (PT-013). However, in contrast to HCPs, several patients reported being cautious about trials that involved changes to their medications: one patient described their body as ‘in a very fragile kind of state’ (PT-008: male/50–59 years/White/failing DDKT) and described being ‘worried doing anything like that [an RCT] would cause me to lose balance again’. Despite patients’ reluctance to have medications changed in a research context, most patients described changes being made to their medications at the time of transplant failure, which were justified to them as aiming to delay failure and prevent infections.

#### Loss and risk aversion

This category was identified in data from both HCPs and patients. Patients’ wishes to maintain the status quo were linked to a strong risk and loss aversion. Patients appeared fearful of research participation that included changes to their medications, and perceived potential losses or harms associated with research participation as greater or more likely than potential benefits, indicating loss aversion. Several patients described not being willing to take part in a trial when the transplant was still functioning but failing. One patient was very specific about only being willing to participate in a drug trial if they had nothing else to lose ‘if you’re dying and you were offered a groundbreaking treatment as a last resort’ (PT-005: male/50–59 years/White/failed DDKT, receiving dialysis). Several HCPs also expressed loss aversion with a preference for trials after a transplant had failed rather than while it was failing. One HCP described being reluctant to recruit to an immunosuppression minimisation trial at the time a transplant was failing because of the risk of worsening the situation: the potential to improve the situation was not explicitly acknowledged.

#### Experiential learning and the availability heuristic

This category was identified in data from HCPs exclusively. In circumstances when HCPs lacked individual equipoise this appeared to relate to memorable cases. This was most evident with respect to transplant nephrectomy after transplant failure, with HCPs reporting witnessing poor outcomes. One HCP described transplant nephrectomy as being risky, resulting in ‘lots of complications’ (HCP-019: male/40–49 years/consultant transplant surgeon) and ‘not straightforward surgery’ (HCP-019). Whilst clinical experience (experiential learning) appeared to explain most HCPs’ views that a nephrectomy RCT was not justified, one HCP did evidence analytical thinking, referring to ‘bad outcomes’ in a published single-centre observational study for people following transplant nephrectomy. As reported earlier, whilst some HCPs acknowledged variation in global practice, and a ‘lack of proof’ for their belief that nephrectomy caused harm, overall HCPs were not willing to support nephrectomy trials. Experiential learning and the availability heuristic also applied to other research studies: one HCP reported a lack of willingness to recruit to a trial comparing stopping the CNI with stopping the anti-proliferative at the time of transplant failure due in part to their clinical experience observing harms (bone marrow suppression and anaemia) if an anti-proliferative is continued. However, the HCP was clear that their willingness to randomise to such a trial depended on the specific patient subgroup, and whether individuals were for retransplantation or not.

### Suggestions for future research

Patients described support for research into the impact of lifestyle factors on transplant survival and outcomes after transplant failure. Suggested trials focussed on aspects of their care that they could control (e.g. diet/exercise) which may suggest a feeling of both a lack of control, a need to control what they can, and a sense of personal responsibility and impact. One HCP, a clinical psychologist, suggested further research to screen and treat for depression at the time of transplant failure and ascertain the best treatment, a view echoed by two patients who suggested evaluating the impact of psychosocial support. One patient described experiencing low mood and described how psychosocial support would have been useful at the time of transplant failure. One HCP, a specialist pharmacist, suggested a trial of SGLT2i medications specifically in the context of transplant failure. The varied perspectives of the different HCPs highlighted the importance of multi-professional teams contributing to the management of and research into transplant failure.

## Discussion

This study provides an in-depth understanding of patient and HCP perspectives on clinical trials to identify best practice for managing kidney transplant failure. To our knowledge this is the only qualitative study investigating this topic. Participants were diverse in terms of age, sex, and transplant type. Our sample included people whose transplants were failing and those whose transplants had failed. HCPs provided diversity of roles in the multidisciplinary team.

### Future trials

This study is part of a programme of mixed-methods research investigating the management of failing transplants and transplant failure. In addition to this qualitative study, we have undertaken a study of national practice patterns [31, 32], and a UK renal registry analysis is underway to investigate centre-variation in outcomes following transplant failure. The overarching goal of our programme of research is to use findings to design one or more RCTs of treatment strategies for failing transplants and kidney transplant failure, to generate high-quality evidence to inform clinical practice [33]. This study found explicitly acknowledged community equipoise regarding the management of immunosuppression, nephrectomy and the use of diagnostic and prognostic investigations, and individual equipoise for immunosuppression management, lifestyle interventions, and the use of diagnostic investigations.

KDIGO’s research agenda highlighted the need to investigate ‘immunosuppression adaptation in the settings of both the failing and failed allograft’: patients and HCPs in our study were most supportive of trials in the *failed* transplant and less supportive of trials at the time a transplant was failing. Trials evaluating therapies to delay graft failure may struggle to recruit to target, and trials starting at the time of transplant failure may be easier to undertake given the challenges in diagnosing and predicting the timing of graft loss. Our study found that HCPs were not supportive of research to address the KDIGO research agenda item to ‘determine the impact of nephrectomy on HLA sensitisation, procedural morbidity/mortality, and inflammation’. Although some HCPs recognised variation in practice globally, all HCPs interviewed appeared to lack individual equipoise regarding nephrectomy.

Missing from the KDIGO’s research agenda were studies evaluating lifestyle interventions (e.g. diet, exercise) which our study found were important to patients; this may indicate such interventions are perceived as ‘low risk’ but also reflected patients’ desire to control something at a time they felt they lacked control [15].

### Precision medicine and clinical trial design

HCPs advocated for stratification within clinical trials, given the heterogeneity of the patient population, with suggested strata defined by age, type/cause of transplant failure, and eligibility for retransplantation. Longitudinal, observational research is needed to further stratify transplant patients using phenotype, clinical history, biopsies, biomarkers, transcriptomics, and genomics.

Conventional clinical trials are unlikely to be feasible to evaluate multiple treatment options in individual patient groups defined by variables such as primary disease, cause of graft failure, age, sensitisation, immune function, immunosenescence, or eligibility for retransplantation. Instead, newer patient-centred trial designs that allow customisation to specific subgroups, such as basket, umbrella and platform trials, are likely to be required. Basket trials study a single therapy in multiple diseases that have common molecular alterations (e.g. SGLT2i trials in failing native and transplant kidneys). Umbrella trials evaluate multiple therapies for a single disease that is stratified into subgroups by molecular alterations (e.g. evaluation of a therapy in subgroups defined by HLA donor-specific antibody sensitisation). Platform trials, also referred to as multi-arm, multi-stage trials, evaluate multiple therapies against a common control group and can be perpetual [34].

### Equipoise

At least half of funded RCTs fail to meet recruitment targets [35] and recruitment difficulties are the most frequent reason for premature trial closure [17, 36]. A lack of individual clinician equipoise is a barrier to RCT recruitment [16–18]. Equipoise is broadly understood as a state of ‘substantial uncertainty’ around treatment superiority. ‘Community equipoise’ describes a lack of consensus in the clinical community about which treatment is best. Most HCPs, but not all, also described personal ‘individual equipoise’ regarding the management of immunosuppression at the time of transplant failure: this finding is encouraging with respect to the potential for undertaking clinical trials in this area. However, communication of clinician uncertainty and equipoise to patients is crucial: patients were reluctant to take part in research that involved changes to their medications, despite medication changes being widespread at the time of graft failure and there being little evidence to inform those changes. Whilst patients and some HCPs expressed a desire to maintain the status quo, it was apparent that the status quo is not leaving medication unchanged at the time of transplant failure, but frequent changes. Ensuring both patients and HCPs have the opportunity to reflect on the current status quo being multiple drug changes with limited evidence to guide decision-making may support recruitment to RCTs.

### Limitations

(i) Family members and friends did not feel able to comment on research and clinical trials in this area. Further work could investigate the roles of relatives in supporting patients’ participation in clinical research; (ii) only one patient was < 40 years old. Future research focussed on the perspectives of children and young adults is required; (iii) there was an assumption in our study that we were talking about graft loss as an irreversible process. Our findings therefore do not apply to the design of research studies to prevent or reverse specific causes of graft loss.

## Conclusions

Our study found HCP and patient support for RCTs after transplant failure, but reticence to engage in trials at the time a transplant was failing (in the 12 months before transplant failure is anticipated). Individual and community equipoise exist to support trial delivery and recruitment. There was strong support for immunosuppression reduction trials, with stratification of people with transplant failure according to clinical and immunological subtypes. Master protocol trials such as umbrella trials may be needed to evaluate therapies in multiple patient subgroups.

## Supplementary Information

Additional file 1.

Additional file 2.

## Data Availability

Anonymised transcripts of the interviews for those participants who consented to data sharing will be uploaded to the University of Bristol’s Research Data Repository: https://data.bris.ac.uk/data/. Audio files of the recorded interviews are not suitable for sharing as they carry a high risk of allowing the research participants to be identified, and the content of interviews includes sensitive information. Individuals who wish to access the dataset can contact the researchers directly. Although the qualitative transcripts have been anonymised, as personal and sensitive issues have been discussed we cannot rule out the risk of identification, and therefore access to these transcripts is controlled. Individual researchers will need to request access to the controlled data through the University of Bristol via the Data Access Committee (DAC) for approval, before data can be shared after their host institution has signed a Data Access Agreement. The procedure for accessing data can be found here: https://www.bristol.ac.uk/staff/researchers/data/accessing-research-data/.

## Abbreviations

CGT: Constructivist grounded theory
CKM: Conservative kidney management
COREQ: Consolidated criteria for reporting qualitative studies
DDKT: Deceased-donor kidney transplant
eGFR: Estimated glomerular filtration rate
HCPs: Healthcare professionals
KDIGO: Kidney Disease: Improving Global Outcomes
LDKT: Living-donor kidney transplant
