# Supplementary material for: Patient and clinician perspectives on clinical trials in transplant failure: a qualitative interview study
Source: Trials. 2026 May 11;27:462. doi: 10.1186/s13063-026-09732-1 (PMC13330387; doi:10.1186/s13063-026-09732-1)
Supplement: Supplementary file 2 — Additional file 2. [file 13063_2026_9732_MOESM2_ESM.docx]

**Reflexivity statement**

The research team was comprised of individuals with diverse personal and professional experiences relevant to the study. As a group, the overall goal was to improve the experiences of people with transplant failure, and improve outcomes following transplant failure: implicit in this was a belief that current care could be improved. All authors believed in the importance of qualitative research to understand lived experiences, identify research questions of importance, identify targets for intervention, and potential care patterns reported to improve experiences and outcomes.

The research group included three practising nephrologists, two with a specialist interest in transplantation and clinical experience of managing the care of people with transplant failure (PKB and SG) and one with a specialist interest in shared-decision making, supporting management choices including conservative kidney management (BH). SG chaired the British Transplantation Society’s (BTS) committee which created the ‘UK Guideline for the management of the patient with a failing kidney transplant’. As part of the guideline preparation, SG engaged in comprehensive reviews of the literature regarding kidney transplant failure. The guidance highlighted gaps in the evidence of management and made recommendations for future research. Prior to this study, SG led a survey of practice patterns regarding the management of kidney transplant failure in hospitals in the UK, and PKB contributed to analysis (findings published). This meant that SG and PKB were aware of the service delivery at potential sites, enabling sites to be purposefully selected to include transplanting and referral hospitals and to include hospitals with difference practice patterns for the management of people with transplant failure. JN trained and practiced as a paediatric nurse before becoming a health services researcher with expertise in applying qualitative and mixed-methods to the development and evaluation of complex interventions. The research group included three medical sociologists (LS, CE, and LH) experienced in the design and delivery of applied qualitative health research projects in kidney disease, transplantation and organ donation. From conception the research team included two people with lived experience of kidney transplantation (AH and PM) and one with multiple experiences of transplant failure (PM). Very sadly, PM died prior to the study starting, but his inputs into the design of the study and the focus of enquiry were invaluable. BV was appointed to the role of research associate for this study after funding was secured. BV has a socio-legal perspective, with a background in legal policy in living kidney donation. BV had no previous familiarity with the research topic, with no experience of transplant failure or prior understanding of the experiences or management of people with transplant failure. Whilst we are all currently based in the UK, the group benefits from individual international experience with previous time living and/or working in Europe, North America, and Africa.

The multidisciplinary nature of the research team strengthened the analytic process. The HCPs provided contextual understanding of managing failing/failed transplants, while the qualitative researchers brought an interpretive distance, enabling critical reflection of everyday clinical practice. Involvement of the patient advisors further allowed assumptions to be challenged and for broadening interpretations of patient and family experiences. Collectively, as a group of investigators we were primarily driven by pragmatism, with goals to identify and address problems in healthcare. We had a collective goal to intervene. The qualitative study was part of a larger mixed-methods programme of work designed to understand the experiences of, management of and outcomes following kidney transplant failure, with a view to developing and evaluating interventions to improve care, experiences and outcomes. We aimed to identify findings that could inform the design and delivery of clinical practice, clinical guidelines, patient and family informational resources and support, and clinical trials.
